# Functional characterization of endocytic signals in the SynDIG/PRRT family members SynDIG1 and SynDIG4 in heterologous cells and neurons

**DOI:** 10.3389/fncel.2024.1526034

**Published:** 2025-01-23

**Authors:** David J. Speca, Chun-Wei He, Christina M. Meyer, Erin C. Scott, Elva Díaz

**Affiliations:** ^1^Department of Pharmacology, School of Medicine, University of California, Davis, Davis, CA, United States; ^2^Max Planck Florida Institute for Neuroscience, Jupiter, FL, United States

**Keywords:** SynDIG1, Capucin, SynDIG4, IFITM3, AP2, GluA1, AMPAR, TGN

## Abstract

The transmembrane protein Synapse Differentiation Induced Gene 4 (SynDIG4), also known as Proline-rich transmembrane protein 1 (PRRT1), is an AMPA-type glutamate receptor (AMPAR) auxiliary factor that is necessary for maintaining extra-synaptic pools of GluA1. Loss of SynDIG4, and the subsequent decrease in extra-synaptic GluA1, has been found to significantly impact synaptic plasticity in the hippocampus. However, how SynDIG4 establishes and maintains these pools is unclear. Previous studies suggested that endocytic machinery is important for maintaining a pool of mobile surface AMPARs, and that proteins associated with such cellular machinery are critical for proper protein trafficking and internalization. Given that SynDIG4 co-localizes with GluA1 in early and recycling endosomes in cultured hippocampal neurons, we sought to identify the sorting signals that target SynDIG4 to endosomes to further elucidate the role of SynDIG4 in GluA1 trafficking. In this study, we report that SynDIG4 possesses a YxxΦ sorting motif, 178-YVPV-181, responsible for binding to the AP-2 complex cargo-sorting subunit μ2. This motif appears critical for proper SynDIG4 internalization, as SynDIG4 mutant 178-AVPA-181, which disrupts binding to μ2, induces aberrant SynDIG4 accumulation at the plasma-membrane of heterologous cells and primary rat hippocampal neurons. We also show that SynDIG4 mutants lacking an endocytic signal co-localize with GluA1 but less so with GluA2 on the surface of heterologous cells. Furthermore, we show that another family member, SynDIG1, is enriched in the trans-Golgi network (TGN) and can traffic between the TGN and plasma membrane. We have identified a non-canonical μ2 binding sequence in SynDIG1 that induces aberrant accumulation at the plasma membrane of heterologous cells and primary rat hippocampal neurons, suggesting a conserved role for μ2-mediated endocytosis within the SynDIG family. These results provide important insight into the mechanisms by which SynDIG proteins are targeted to endosomal compartments as a step in understanding SynDIG-mediated regulation of AMPAR trafficking.

## Introduction

The founding member of the *Synapse Differentiation Induced Gene* (*SynDIG*) family of AMPA receptor (AMPAR) auxiliary factors was originally identified in a microarray screen for molecules differentially expressed during the period of synapse development in the mouse cerebellum (Díaz et al., 2002). Functional studies of *SynDIG1*, which is also expressed in the hippocampus and throughout the cortex, demonstrated that SynDIG1 regulates AMPAR content at developing synapses in dissociated rat hippocampal neurons (Kalashnikova et al., 2010). Furthermore, SynDIG1 was found to positively influence excitatory synapse number when overexpressed in cultured hippocampal neurons. Ultrastructural analysis of hippocampal synapses in mice lacking SynDIG1 showed a decrease in the number of synapses with a mature phenotype (Chenaux et al., 2016). In a separate study using hippocampal slice preparations, it was demonstrated that while SynDIG1 did not influence AMPAR biophysical properties, it nonetheless increases both AMPAR and NMDA receptor (NMDAR) content and functional excitatory synapse number when overexpressed (Lovero et al., 2013). Taken together, these studies suggest that SynDIG1 is involved in excitatory synapse development and maturation. Intriguingly, loss of SynDIG1 also blocks homeostatic synaptic plasticity in response to tetrodotoxin treatment (Kaur et al., 2016), and indeed it has been shown that SynDIG1 is phosphorylated in response to treatment with tetrodotoxin (Wu et al., 2022).

In contrast to SynDIG1, we found that a related family member, SynDIG4 does influence biophysical properties of AMPARs in a subunit-dependent manner. Specifically, coexpression of SynDIG4 with GluA1 or GluA1 + GluA2 in *Xenopus* oocytes slows their deactivation kinetics (Matt et al., 2018), indicating that SynDIG4 interacts directly with AMPARs. In support of this finding, several studies have identified SynDIG4 in GluA1-containing AMPAR complexes, including mass spectrometry studies of affinity-purified AMPARs (Shanks et al., 2012; Schwenk et al., 2012; Chen et al., 2014) and cryo-electron microscopy (cryoEM) performed on native AMPARs isolated from mouse brain tissue (Yu et al., 2021). Electrophysiological recordings in hippocampal slices from mice lacking SynDIG4 reveal deficits in one form of long-term potentiation (1 x 100 Hz tetanus LTP) (Matt et al., 2018) and in long term depression (LTD) (Troyano-Rodriguez et al., 2019). In addition to biophysical changes, there is reduced surface expression of GluA1 and GluA2 in hippocampal lysates (Troyano-Rodriguez et al., 2019) and a striking deficit in extrasynaptic GluA1 and GluA2 in cultured hippocampal neurons from mice lacking SynDIG4 (Matt et al., 2018), suggesting that SynDIG4 also plays a role in trafficking or stabilization of AMPARs.

Bioinformatic analysis has revealed that the SynDIGs share a distant structural homology with at least 10 other molecules (Sällman Almén et al., 2012), including a family of four molecules named Interferon Inducible Transmembrane proteins (IFITMs), which are involved in the innate immune response (Zhao et al., 2018; Everitt et al., 2012). The larger superfamily was originally designated “Dispanins” because of two predicted helical hydrophobic segments capable of spanning the plasma membrane. However, subsequent studies have shown that they are single-pass type II transmembrane proteins with internal N-termini and extracellular C-termini (Kalashnikova et al., 2010; Kirk et al., 2016; Ling et al., 2016; Martin et al., 2021; Weston et al., 2014). The first helix, rather than spanning the membrane, may be buried in the membrane, and in the case of IFITM3, this helix has amphipathic character which may associate with the inner leaflet of the plasma membrane (Ling et al., 2016; Chesarino et al., 2017). In addition to the shared topology, dispanin family members share two conserved cysteine residues which can be reversibly palmitoylated (Kaur et al., 2016; Yount et al., 2010; Yount et al., 2012). It has been proposed that the function of this family of molecules is to act as “fusogens” by either promoting or inhibiting membrane fusion (Coomer et al., 2021).

IFITM3 is trafficked transiently to the plasma membrane but rapidly endocytosed and transported to the endolysosomal network, where it acts to restrict viral entry, perhaps by inhibiting the formation of a fusion pore (Spence et al., 2019; Desai et al., 2014). IFITM3 harbors an endocytic signal which interacts with the μ2 subunit of the multi-subunit Adaptor Complex, AP2 (Jia et al., 2014; Chesarino et al., 2014). This complex forms a bridge between cargo and clathrin and is critical for clathrin-mediated endocytosis (Bonifacino and Traub, 2003). Mutation of this endocytic signal disrupts the interaction with μ2, resulting in accumulation of IFITM3 on the plasma membrane and an alteration in the ability of IFITM3 to restrict viral entry (Jia et al., 2014; Chesarino et al., 2014).

This work inspired us to explore whether the SynDIGs also harbor endocytic signals which could interact with *μ*2 and the μ subunits of other adaptor complexes which are involved in many different intracellular trafficking pathways (Guardia et al., 2018). A better understanding of the trafficking of the SynDIGs might provide greater insight into how SynDIG4 and SynDIG1 can influence AMPAR trafficking and synapse development.

## Materials and methods

### Animals and neuronal culture

All animal use was approved by the institutional animal care and use committee at the University of California, Davis, in accord with the guidelines laid out by the US Public Health Service. For rat cultures, hippocampal neurons were dissected from wild-type E18 Sprague Dawley rat pups of both sexes and plated in 6-well plates at a density of 150,000 cells per well. For mouse cultures, hippocampal neurons were dissected from wild-type P0 C57BL/6J mouse pups of both sexes and plated in 6-well plates at a density of 100,000 cells per well. Cells from both cultures were maintained in wild-type rat-derived astrocyte-conditioned neuron maintenance medium (NMM) consisting of 1X Neurobasal (NB) medium (Gibco #21103049), 1X GlutaMAX (Gibco #35050061), and 1X B-27 supplement (Gibco #17504044). Rat cultures were transfected with the indicated plasmids using the subsequently detailed protocol between 5 and 9 days *in vitro* (DIV). Mouse cultures were transfected with the indicated plasmids using the same protocol at DIV9.

For transfection, NB medium was equilibrated in the incubator for at least 1 h. Then, the coverslips were transferred into the equilibrated NB. Concurrently, 1.5 μg of DNA in 50 μL of NB was mixed with 2.5 μL of Lipofectamine 2000 (Invitrogen #11668027) in 50 μL of NB. This mixture was incubated at room temperature (RT) for 35 to 60 min. The Lipofectamine:DNA mix was then added to the neurons and allowed to incubate in a 37°C incubator for 1 h before being transferred back to the astrocyte-conditioned NMM.

For rat cultures, DIV18 ~ 22 hippocampal cultures were fixed with 4% paraformaldehyde (PFA) and 4% sucrose in PBS for 5 min at RT. Coverslips were washed once with cold PBS followed by blocking with 5% fetal bovine serum in PBS (blocking solution) at RT for 2 h. The neurons were stained with the anti-HA antibody (Cell Signaling Technology Cat# 3724, RRID: AB_1549585) at RT. After 2 h, the neurons were then washed three times with PBS, followed by permeabilization with 0.25% Triton X-100 at RT for 8 min. Coverslips were washed with PBST (0.01% Triton in PBS) and incubated with blocking solution at RT for 2 h. The antibodies against PSD95 (UC Davis/NIH NeuroMab Facility Cat# K28/43, RRID: AB_2877189) and FLAG (Sigma-Aldrich Cat# F3165, RRID: AB_259529) were added to the neurons at 4°C overnight. After washing three times with PBST, coverslips were incubated in blocking solution at RT for 2 h and fluorophore-conjugated secondary antibodies were applied to the coverslips for 2 h at RT.

For mouse cultures, DIV18 hippocampal cultures were live-fed the previously listed anti-Flag antibody for 10 min at RT, followed by a 10-min incubation of the secondary antibody (Invitrogen Goat anti-Mouse IgG1 Alex Fluor 647 Cat#A-21240; RRID: AB_2535809). Cultures were then fixed with 4% PFA and 4% sucrose in PBS for 10 min at RT, followed by three washes in RT PBS. All subsequent steps follow the rat culture protocol, with the difference being that anti-myc (Cell Signaling Cat#9B11, RRID: AB_331783) and MAP2 (Millipore Cat#AB5622-I, RRID: AB_2800501) antibodies were added to neurons at 4°C overnight.

### Plasmids

Epitope tags were added, or amino acid substitution mutations were made to cDNAs from mouse SynDIG1, mouse SynDIG2 or rat SynDIG4 using a Q5® Site-directed mutagenesis kit (NEB Cat#E0554S). For N-terminal tags, a methionine codon was added to the 5′ end of the sequence encoding the epitope tag and the original methionine start codon was replaced with this sequence. For C-terminal epitope tags, the epitope tag sequence was inserted directly after the final codon. For C-terminally tagged SynDIG1-Flag and associated deletion mutants, a flexible linker was added to facilitate proper expression (sequence: GGSGGDYKDDDDK). Information on all constructs used in this study are described in greater detail in Supplementary Table 1.

### Cell culture

All cell lines were grown in a humidified incubator at 37°C and 5% CO_2_. COS-7 cells (ATCC Cat# CRL-1651, RRID: CVCL_0224) and HEK293T cells (ATCC Cat# CRL-3216) were maintained in Dulbecco’s modified Eagle’s medium (Gibco Cat # 11995–065) supplemented with 10% fetal bovine serum (Avantor Seradigm Cat#97068–085) and 1% penicillin/streptomycin.

### Co-immunoprecipitation

For co-immunoprecipitation studies, HEK293T cells were used because of their robust transfection and transient expression ability. On day one, 170,000 cells/well were seeded into 6 well plates. On day three, confluent cells were transfected for 4 h with 2.5 μg DNA/well (500 ng SynDIG +2,000 ng μAP subunit or pcDNA3.1 empty vector) using Lipofectamine 2000 (ThermoFisher Cat# 11668027), and cells were allowed to express the constructs for >24 h. On day four, co-immunoprecipitation was performed using an α-HA magnetic bead immunoprecipitation (IP) kit (ThermoFisher Cat# 88838) following the manufacturer’s protocol. In brief, cells were rinsed twice with ice cold phosphate-buffered saline (PBS) containing 1 mM CaCl_2_, 0.5 mM MgCl_2_ and solubilized with 500 μL IP wash buffer/well (including protease inhibitors and PMSF) for 90 min at 4°C with rotation. Lysates from two wells were combined for each replicate (1,000 μL total). Nuclei and insoluble material were pelleted with centrifugation at >13,000xg for 15 min at 4°C. 80 μL of solubilized protein was reserved as input, while 800 μL of supernatant was added to 30 μL α-HA magnetic beads and incubated for 90 min at RT with rotation. Beads were then washed three times with IP wash buffer, once with double-distilled H_2_O and then eluted with 100 μL non-reducing sample buffer (NRSB). Dithiothreitol (DTT, 50 mM) was added following elution.

### Immunoblotting

Input (15 μL) and IP (50 μL) protein samples were heated to 70°C for 10 min and separated via gel electrophoresis on a Bio-Rad minigel system using 4–20% gradient gels (Bio-Rad Cat# 456–1,094). Following separation and transfer to nitrocellulose, membranes were blocked with 4% milk in Tris-buffered saline (TBS) with 0.1% Tween-20 (TBST) for 60 min at RT. Membranes were then incubated overnight at 4°C with the following primary antibodies in 4% TBST: Mouse α-myc IgG2a (Cell Signaling Technology Cat# 2276, RRID:AB_331783, 1:1,000), Rat α-HA (Roche Cat# 11867423001, RRID:AB_390918, 1:500, for use on input samples) or Rabbit α-HA (Cell Signaling Technology Cat# 3724, RRID:AB_1549585, 1:1,000, for use on IP samples). The following day, membranes were washed three times with TBST and incubated with the following secondary antibodies: Goat α-mouse Azure 700 (Azure Biosystems Cat# AC2129, RRID:AB_3331665, 1:1,000) and Goat α-Rat H + L Alexa 488 (Jackson ImmunoResearch Labs Cat# 112–545-167, RRID:AB_2338362; 1:500, for use on input samples) or Goat α-Rabbit Alexa 790 (Jackson ImmunoResearch Labs Cat# 111–655-144, RRID:AB_2338086; 1:500, or use on IP samples). Membranes were washed three times with TBST and once with TBS prior to imaging.

### Imaging and quantification of immunoblots

Fluorescent immunoblot images were acquired on a Sapphire Bioimager (Azure Biosystems Model #Sapphire RGBNIR) and quantified with Azure spot software (ver 2.0). For analysis, the amount of eluted interacting protein (in most cases, a myc-tagged SynDIG construct) was normalized to both the amount of eluted interacting protein (in most cases, an HA-tagged μ2 subunit) and to the amount of input interacting protein (in most cases, a myc-tagged SynDIG construct).

### Immunofluorescence immunocytochemistry

For immunocytochemistry experiments, COS7 cells were used to better visualize intracellular organelles. For each experiment, 30,000 cells/well were seeded onto coverslips coated with collagen in 6 well tissue culture plates. The following day, nonconfluent cells were transfected for 4 h with 1 μg DNA (250 ng SynDIG +750 ng empty vector) using Lipofectamine 2000. The following day, transfected cells were fixed and stained; however, prior to fixation, the following procedures were used: for live **surface labeling** experiments, cells were rinsed twice with ice cold PBS (containing 1 mM CaCl_2_, 0.5 mM MgCl_2_) and then blocked for 20 min with prechilled 0.2% bovine serum albumin (BSA) in PBS at 4°C. Cells were then incubated for 20 min with primary antibodies [either Mouse α-myc IgG2a (Cell Signaling Technology Cat# 2276, RRID:AB_331783, 1:1,000), Rat α-HA (Roche Cat# 11867423001, RRID:AB_390918, 1:200), or Ms. α-Flag (Sigma-Aldrich Cat# F3165, RRID: AB_259529; 1:500)], followed by three BSA-PBS washes and incubation with appropriate secondary antibodies. For **antibody feeding** experiments, myc-SynDIG1-Flag transfected cells were incubated with α-Flag antibody at 37°C for 60 min. For **steady state localization** experiments, cycloheximide (100 μM) was added to the media for four hours prior to fixation at 37°C to inhibit protein translation.

Regardless of treatment, all cells were then rinsed three times with ice cold PBS (+1 mM CaCl_2_, 0.5 mM MgCl_2_) and fixed with prechilled 3% glyoxal solution (Richter et al., 2018) for 30 min at RT, followed by one rinse with PBS, quenching with ammonium chloride (50 mM) for 15 min, permeabilization with 0.1% Triton X-100 and blocking with BSA for 60 min (all diluted in PBS). Cells were incubated overnight at 4°C with primary antibodies to Golgin-97 (Cell Signaling Technology Cat# 13192, RRID: AB_2798144, 1:100), EEA1 (BD Biosciences Cat# 610456, RRID: AB_397829; 1:250), GM130 (BD Biosciences Cat# 610822, RRID: AB_398141; 1:500), and α-myc or α-HA antibodies noted above.

### Confocal imaging and analysis

Images were acquired on a Leica SP8 instrument in confocal mode with a 63x objective and z stack images were taken through the entire cell volume. Image analysis was performed using FIJI/ImageJ (ver. 2.14.0/1.54f). For determination of surface expression, we analyzed maximum projections. Regions of interest (ROI) were drawn manually around the border of individual cells and integrated density (using a uniform threshold determined for each channel) was measured for both surface and total expression and used to generate a ratio. The SynDIG2 constructs only had a C-terminal HA tag. The total stain integrated density for the SynDIG2∆135–55 deletion mutant was very low, perhaps because the HA epitope recognized by the α-HA antibody used for labeling total protein was blocked by the α-HA antibody used for surface staining. This led to unrealistically high surface/total ratios. To produce a more realistic value for total stain, we added surface+total to create a total stain value to be used as the denominator. To calculate Manders Correlation Coefficients (MCC), we used JaCoP plugin, using manually determined thresholds. For MCC calculations to improve resolution of GM130 and Golgin-97, zoomed in images were acquired and deconvoluted using Huygens Software (Scientific Volume Imaging), and a single z slice was used for analysis. For analysis of SynDIG4 overlap with surface AMPARs, to improve resolution, images were deconvolved and maximum projections of surface staining were used for analysis. In this situation a uniform square ROI was centered over the cell to avoid cell edges which tended to have artefactual staining.

### Statistics

Statistical analysis was performed using GraphPad Prism (ver. 10.3.1). In experiments where two groups were compared, we used an unpaired *t*-test, except for the calculation of GM130 and Golgin-97 co-localization where we used a paired t-test. In experiments comparing three or more groups, if the variance was not significantly different, we used a one-way ANOVA with post-hoc Tukey test. If the variance was significantly different, we used a Brown-Forsythe ANOVA with post-hoc Dunnett’s test.

## Results

### SynDIGs associate with μAP subunits

**Figure 1:**
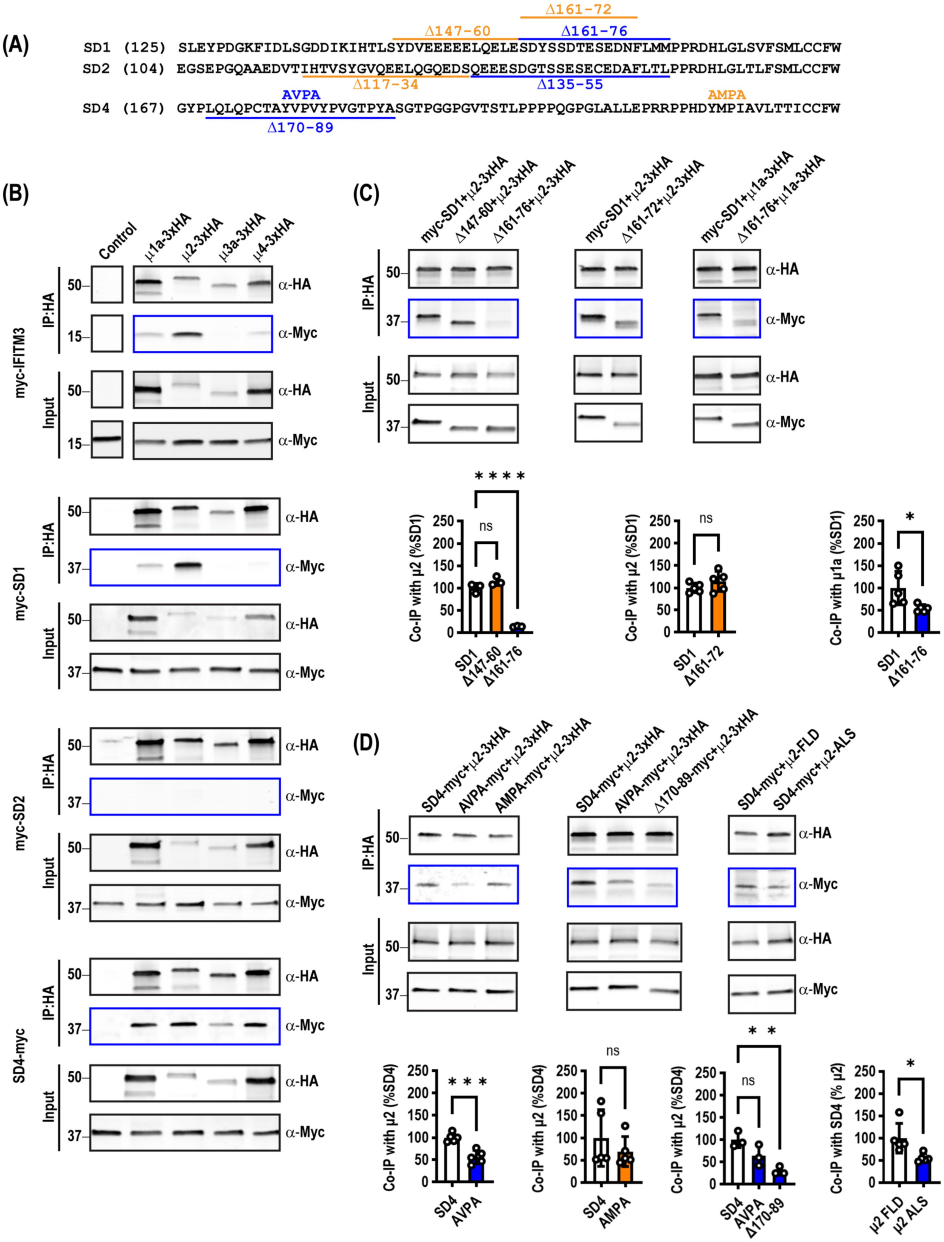
SynDIG1 and SynDIG4 co-immunoprecipitate with the μ2 AP subunit. HA-tagged μAP subunits were used to co-immunoprecipitate myc-tagged SynDIG constructs expressed in HEK293T cells. **(A)** Schematic diagram of SynDIG deletion mutants used in this study. Number in parentheses indicates position of the leftmost amino acid. Constructs highlighted in blue disrupted the interaction with μ2 and/or influenced surface expression whereas constructs highlighted in orange did not. **(B)** Representative survey of interactions between μAP subunits and SynDIGs. **(C)** Representative images and quantification of interactions between SynDIG1 and μ2 and μ1a. **(D)** Representative images and quantification of interactions between SynDIG4 and μ2. To calculate co-immunoprecipitation values, the amount of SynDIG protein immunoprecipitated was divided by the amount of μ protein immunoprecipitated and then normalized to the amount of SynDIG protein in the input. The mean value of all the control samples was then used to normalize all the values within a given experiment. Data are represented as mea*n* ± SEM; *n* = 3–5 biological replicates per group; ns, not significant. *p < 0.05; **p < 0.01; ***p < 0.001; ****p < 0.0001.

Inspired by research into the endocytic trafficking of IFITM3 mediated by an interaction with the μ2 AP subunit (Jia et al., 2014; Chesarino et al., 2014), we performed co-immunoprecipitation experiments in HEK293T cells with several SynDIG proteins: SynDIG1 (also known as Tmem90b), the closely related SynDIG2 (also known as SynDIG1L, Tmem90a, or capucin) and SynDIG4 (also known as Prrt1 or NG5) (Figure 1). We used IFITM3 as a positive control for interaction with μ2 and surveyed multiple μAP subunits, including μ1a, μ2, μ3a and μ4. We confirmed that IFITM3 co-immunoprecipitates robustly with μ2 as previously reported (Jia et al., 2014), but we also detected weaker interactions with μ1a and μ4. We found that SynDIG1 interacted strongly with μ2 and to a lesser extent with μ1a. SynDIG4 was differentially co-immunoprecipitated by all four μAP subunits, while SynDIG2 did not appear to interact with μ2 or any μAP subunit, despite being closely related to SynDIG1 (43% identity, 53% similarity) (Figure 1B). We searched for canonical endocytic signals including tyrosine-based YXXɸ (ɸ being a bulky hydrophobic amino acid) or dileucine-based motifs (Bonifacino and Traub, 2003). We found no canonical signals in SynDIG1; however, several large deletions of the intracellular domain led us to focus on amino acids 144–180 (C-W.H. & D.J.S., unpublished observations) (Figure 1A), and we made three deletion mutants within this critical region for co-immunoprecipitation and further studies. We found that deletion of amino acids 161–76 eliminated almost all binding to μ2 (*F* = 127.5*, p* < 0.0001) (Figure 1C). Interestingly, a smaller deletion of residues 161–72 still retains binding to μ2 when normalized for its lower total expression, suggesting that amino acids 173-FLMM-176 could be a noncanonical endocytic signal with a phenylalanine residue instead of a tyrosine. Furthermore, we observed significantly decreased binding of the 161–76 deletion to the *μ*1a subunit (Figure 1C).

The intracellular portion of SynDIG4 has two potential YXXɸ signals, 178-YVPV-181 and 223-YMPI-226 (Figure 1A). We made alanine substitution mutants (YVPV→AVPA and YMPI→AMPA) to test for co-immunoprecipitation with μ2. We found that the YVPV→AVPA mutant significantly decreased the interaction with μ2 while the YMPI→AMPA did not; however, it was clear that the interaction was not eliminated completely. Therefore, we made a deletion mutation of amino acids surrounding the YXXɸ signal, and this further decreases the interaction (*F* = 11.38, *p* = 0.0091) (Figure 1D). The crystal structures of μ subunits reveal critical conserved amino acids that bind to the tyrosine residue in the YXXɸ signal (Heldwein et al., 2004; Owen and Evans, 1998). We mutated two of these residues (FLD → ALS) and showed that less SynDIG4 protein is co-immunoprecipitated with the μ2 ALS mutant, suggesting that the SynDIG4 signal is binding in the same pocket (Figure 1D).

### Mutation of μ2AP binding sites in SynDIGs increases surface expression

**Figure 2:**
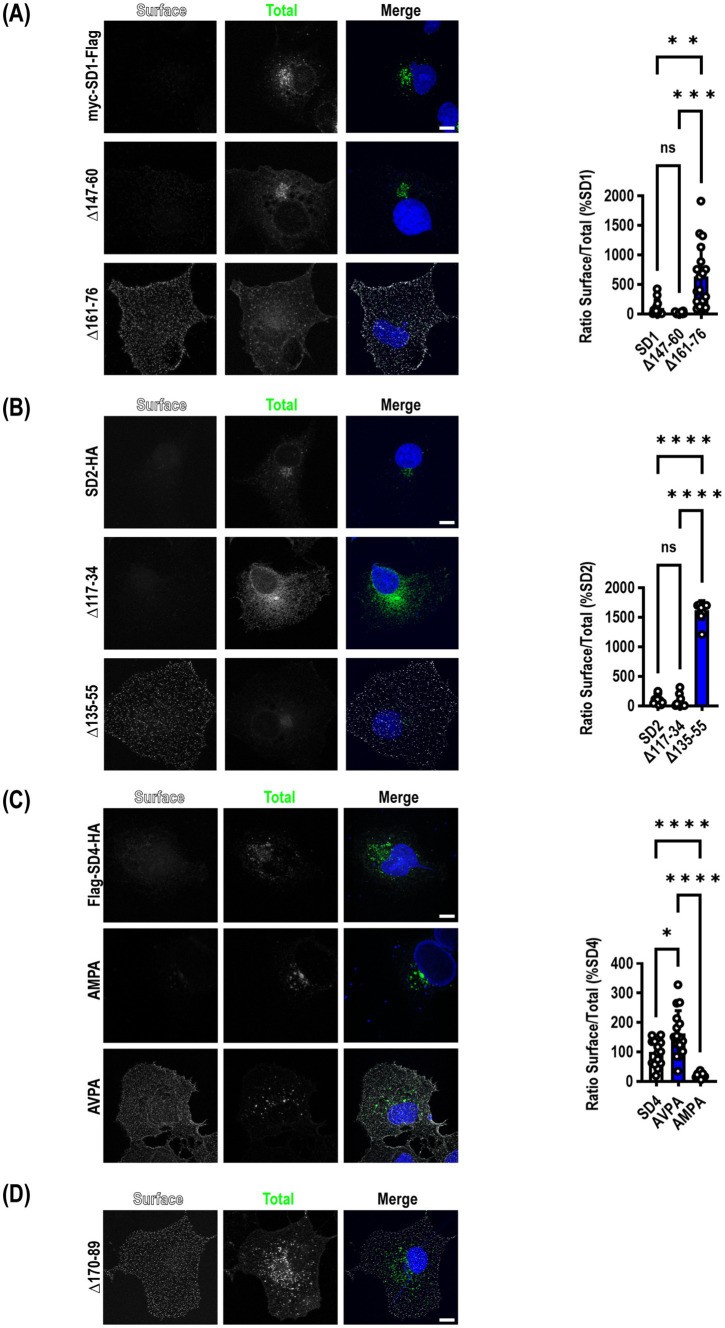
Mutation of μ2AP binding sites in SynDIGs increases surface expression. SynDIG constructs with C-terminal extracellular epitope tags were expressed in COS7 cells and surface labelled. **(A)** Representative images and quantification of surface expression of SynDIG1 and mutants. **(B)** Representative images and quantification of surface expression of SynDIG2 and mutants. **(C)** Representative images and quantification of surface expression of SynDIG4 and mutants. **(D)** Representative image of SynDIG4∆170–89 deletion mutant. Data are represented as mea*n* ± SEM; *n* = 13–20 cells per group **(A)**; *n* = 10 cells per group **(B)**; *n* = 13–16 cells per group **(C)**, each group from 2 to 3 independent experiments; ns not significant; *p < 0.05; **p < 0.01; ***p < 0.001; ****p < 0.0001. Scale bar: 10 μm.

The same deletion constructs tested for their ability to co-immunoprecipitate with μ2 were transfected into COS7 cells to determine whether mutants that disrupted an interaction with μ2 resulted in increased accumulation of SynDIGs on the plasma membrane, as would be expected when endocytosis is disrupted. Cells were live labeled on ice with appropriate primary and secondary antibodies to recognize an extracellular epitope tag, then fixed and stained for total expression of the same molecule. For SynDIG1, we observed very little wild type (WT) protein expressed on the surface, but there was a significant increase in surface expression of the 161–76 deletion mutant that eliminates the interaction with μ2 (*F* = 20.97, *p* < 0.0001) (Figure 2A).

Although we did not detect an interaction between μ2 and SynDIG2, we included several deletion mutants in our surface expression studies for comparison. This was done in part to exploit the high conservation between SynDIG1 and SynDIG2 to map the noncanonical endocytic signal in SynDIG1 (Figure 1A). Like SynDIG1, we observed very little surface expression of WT SynDIG2; however, to our surprise, we observed a significant increase in surface expression of the SynDIG2 135–55 deletion mutant, which is roughly equivalent to the SynDIG1 161–76 deletion mutant (*F* = 549.8, *p* < 0.0001) (Figure 2B,). This suggests that SynDIG2 has the capability to traffic to the plasma membrane, but that accumulation on the cell surface does not depend on a disruption in binding to μ2. We propose several possible explanations in the discussion section.

We quantified surface expression of WT SynDIG4 and the two endocytic mutants and found a significant increase in AVPA surface expression relative to WT, as we hypothesized, but we also observed decreased surface expression of the AMPA mutant relative to WT (*F* = 27.82, *p* < 0.0001) (Figure 2C). This could be due to a defect in protein folding. Alternatively, it is possible that the AMPA mutant disrupts binding to another μ protein such as μ1a, which could interfere with forward trafficking. In an independent experiment, we confirmed that the SynDIG4 170–89 deletion mutant accumulates on the plasma membrane (Figure 2D).

### SynDIG1 and SynDIG2 expression is enriched in the trans-Golgi network (TGN) and traffic between the plasma membrane, early endosomes and the TGN in COS7 cells

**Figure 3:**
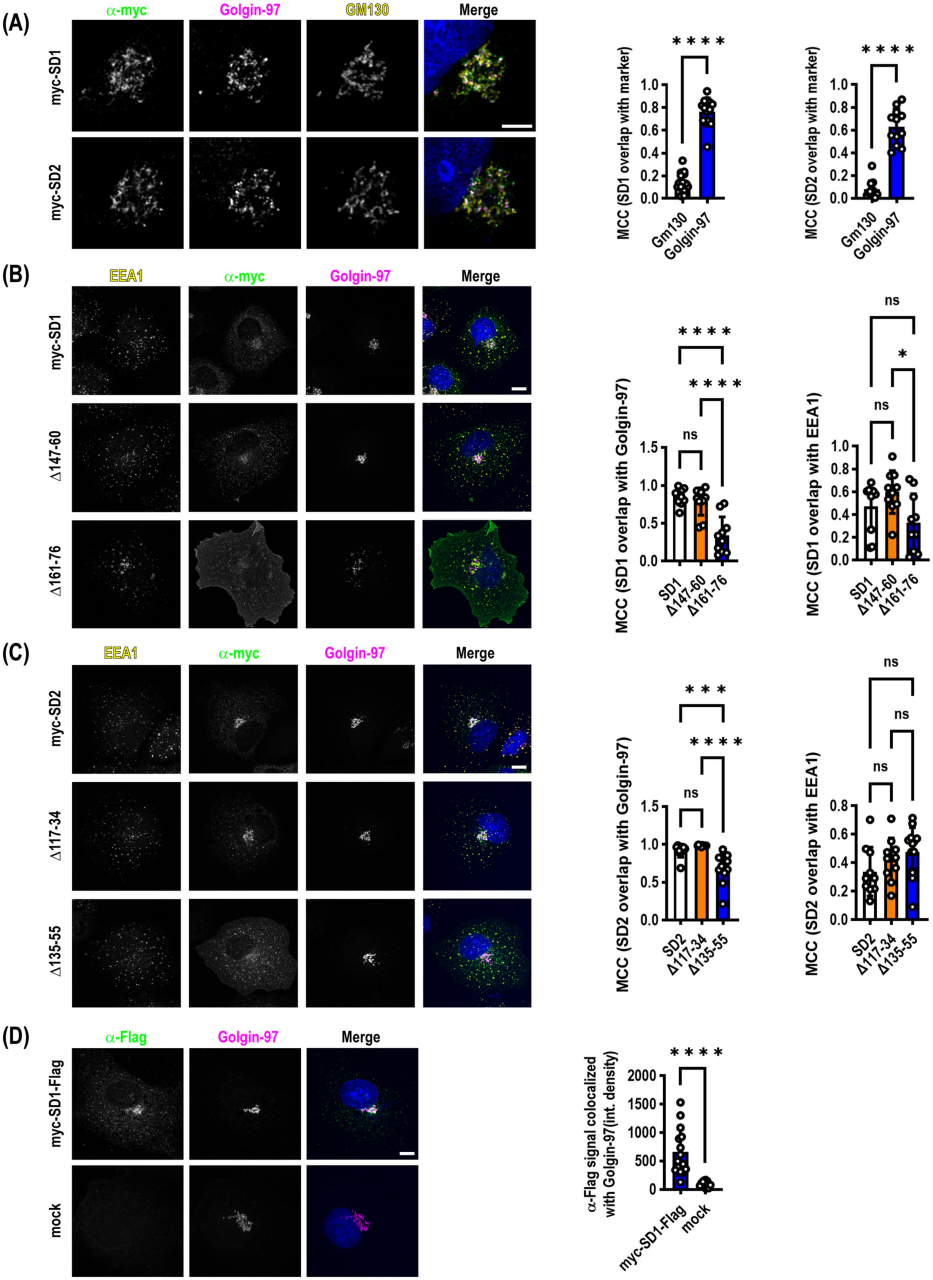
SynDIG1 and SynDIG2 are enriched in the trans-Golgi network. SynDIGs were transiently transfected into COS7 cells to investigate steady state localization after cycloheximide-mediated protein synthesis inhibition. **(A)** Representative images and quantification of SynDIG1 and SynDIG2 co-localization with cis- (GM130) and trans-Golgi (Golgin-97) markers. **(B)** Representative images and quantification of colocalization of SynDIG1 mutants with Golgin-97 and EEA1. **(C)** Representative images and quantification of colocalization of SynDIG2 mutants with Golgin-97 and EEA1. **(D)** SynDIG1 transfected cells (without cycloheximide treatment) were incubated with α-Flag antibody for 1 h at 37°C to assess trafficking from the plasma membrane to the TGN. MCC, Manders Correlation Coefficient. Data are represented as mea*n* ± SEM; *n* = 14 cells for SynDIG1; *n* = 13 cells for SynDIG2 **(A)**; *n* = 10 cells per group **(B)**; *n* = 10–13 cells per group **(C)**; *n* = 12–15 cells per group **(D)**, each group from 2–3 independent experiments; ns not significant; *p < 0.05; **p < 0.01; ***p < 0.001; ****p < 0.0001. Scale bar: 10 μm.

Previous studies indicated that SynDIG2--also named capucin because of its pronounced expression within the caudate putamen--is localized to the cis-Golgi when transiently expressed in HeLa or CHO tissue culture cells (de Chaldée et al., 2006). We reproduced this finding in transiently transfected COS7 cells that were allowed to express for >24 h followed by the addition of 100 μM cycloheximide for 4 h to inhibit protein translation to investigate steady state localization of both SynDIG2 and SynDIG1. We also observed an enrichment in the Golgi; however, when we quantified the colocalization of SynDIG1 and SynDIG2 with cis-Golgi (GM130) and trans-Golgi (Golgin-97) markers, we observed a highly statistically significant enrichment of both proteins in the TGN as opposed to the cis-Golgi (Figure 3A).

We performed the same cycloheximide-based steady state analysis with the SynDIG1 and SynDIG2 deletion mutants. In both cases, we observed a statistically significant decrease in the correlation coefficient between the TGN marker and the deletion mutants which accumulate at the plasma membrane, compared to WT (SynDIG1: *F* = 22.5, *p* < 0.0001; SynDIG2: *F* = 19.37, *p* < 0.0001). We also quantified correlation coefficients for the early endosome marker EEA1 with these same molecules, demonstrating that at steady state, these molecules are also localized with early endosomes (Figures 3B,C). SynDIG4 and IFITM3 do not localize to the TGN under steady state conditions, rather they both localize to early endosomes and lysosomes (D.J.S., unpublished observations).

To investigate whether WT SynDIG1 can traffic to the plasma membrane transiently, be endocytosed, and then be returned to the TGN, we performed an antibody feeding experiment. We incubated transfected cells with primary α-Flag antibody for 1 h at 37°C, then washed, fixed and stained for Golgin-97 and labelled with secondary antibodies. We quantified the integrated density of the α-Flag signal that overlaps with Golgin-97 and determined there was a highly significant increase in signal relative to mock transfected cells, which had very little internalized signal (Figure 3D).

Taken together, these data suggest that SynDIG1 and perhaps also SynDIG2 are enriched in the TGN and can be transported to the plasma membrane where they are rapidly endocytosed and trafficked back to the TGN, although other trafficking pathways are possible.

### An endocytosis defective SynDIG4 mutant co-localizes with GluA1 at the plasma membrane in COS7 cells

Previously, we have shown that co-transfection of WT SynDIG4 with GluA1 can increase the size of GluA1 clusters in COS7 cells (Plambeck et al., 2022). However, because very little SynDIG4 is present on the plasma membrane, these clusters are not located on the cell surface. In fact, they only occur when surface HA-GluA1 is labelled with primary antibody and then incubated at 37°C for 20–30 min, allowing the receptor to internalize. A similar increase in cluster size is also observed when SynDIG4 is co-transfected with GluA2, but not when co-transfected with GluK2 (Plambeck et al., 2022).

**Figure 4:**
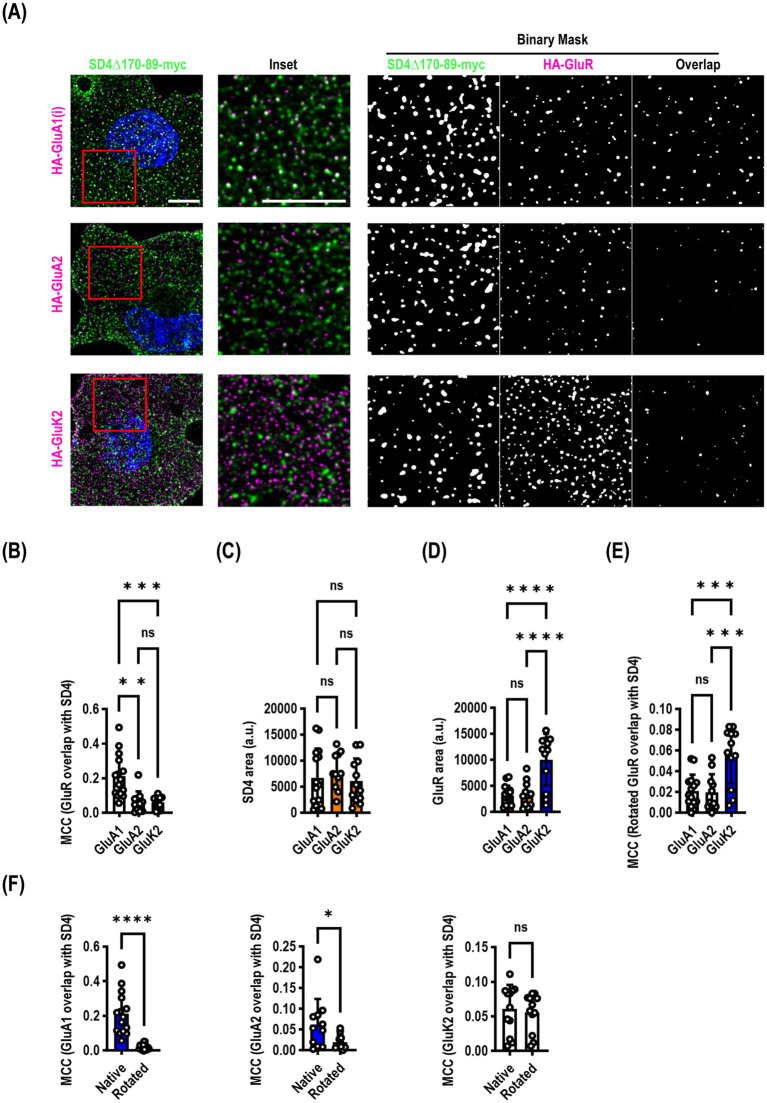
SynDIG4 can colocalize with GluA1 at the plasma membrane. COS7 cells were transiently transfected with SynDIG4∆170-89-myc and HA-tagged ionotropic glutamate receptors and analyzed for colocalization of surface signal. **(A)** Representative images of SD4∆170–89 colocalization with HA-GluA1, HA-GluA2 and HA-GluK2. **(B)** SynDIG4 colocalization with GluA1 is significantly greater that with GluA2 or GluK2. This is not due to differences in SynDIG4 expression **(C)** or increased GluA1 expression **(D)**. As an additional control **(E)**, to randomize the data, the SynDIG4 channel was rotated 90 degrees, and the Manders correlation coefficient (MCC) was recalculated. There was no difference between GluA1 and GluA2; however, the GluK2 MCC was significantly higher, likely due to the higher surface expression of GluK2. **(F)** There was a significant difference in the MCC between native and randomized data for GluA1 and GluA2, but not GluK2. Data are represented as mea*n* ± SEM; *n* = 11–15 cells per group, each group from 2–3 independent experiments; ns not significant; *p < 0.05;**p < 0.01; ***p < 0.001; ****p < 0.0001. Scale bar: 10 μm.

Our endocytosis deficient SynDIG4 mutants enabled us to ask a slightly different question: does SynDIG4 co-localize with ionotropic glutamate receptors at the surface of the plasma membrane? We co-transfected SynDIG4∆170-89-myc with either HA-GluA1 (flip), HA-GluA2, or HA-GluK2 and live surface labelled both molecules. We found that there was a significantly higher Manders correlation coefficient (MCC) between SynDIG4 and GluA1 than either GluA2 or GluK2 (*F* = 16.02, *p* < 0.0001) (Figures 4A,B). We calculated the surface area of the puncta and observed no difference in SynDIG4 when co-expressed with each receptor (Figure 4C); however, GluK2 was expressed at a consistently higher level than GluA1 or GluA2 (Figure 4D). We rotated the SynDIG4 channel to randomize the data and recalculated the MCC (Figure 4E) and found a significantly higher MCC for GluK2, which was likely driven by its higher expression. Comparing native versus rotated (randomized) MCC analysis between SynDIG4 and GluR constructs, we observed a highly significant difference for GluA1, a significant difference for GluA2 and no difference for GluK2 (Figure 4F).

We were somewhat surprised, based on our previous findings, that GluA2 did not demonstrate a higher correlation coefficient. It is possible that SynDIG4 does not co-localize with GluA2 at the plasma membrane, but it is important to note that the construct used for this experiment lacked 20 amino acids which could be important for mediating interactions with other molecules, including GluA2.

### Endocytosis defective SynDIG mutations promote increased surface expression in cultured hippocampal neurons

**Figure 5:**
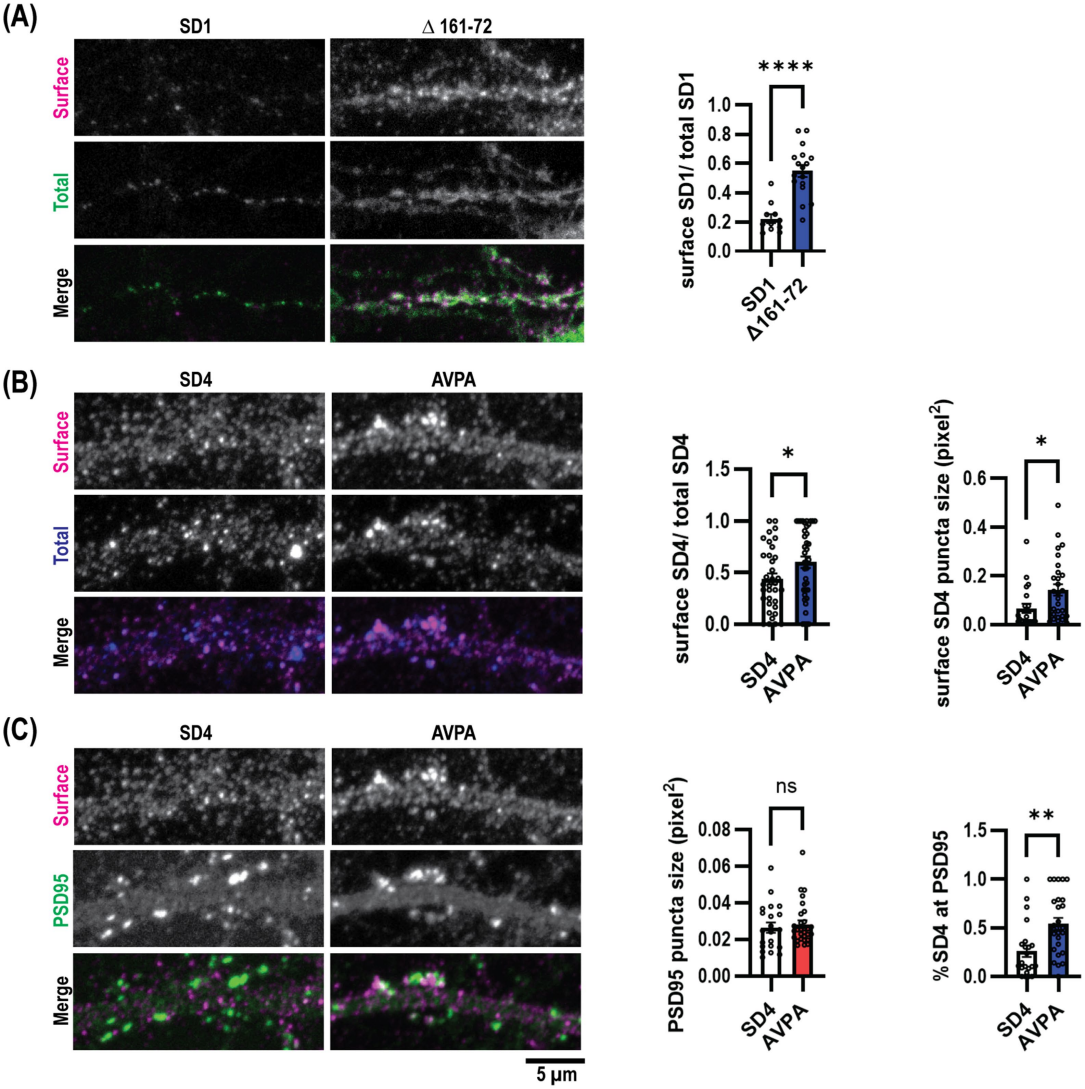
Endocytosis defective SynDIG mutations promote increased surface expression in cultured hippocampal neurons. Cultured hippocampal neurons were transfected and stained for surface and total SynDIG expression. **(A)** Representative dendrites with wild-type SynDIG1 and SynDIG1∆161–76, and quantification of the ratio of surface-labeled to total-labeled SynDIG1 for wild-type and SynDIG1∆161–76. **(B)** Representative dendrites with wild-type SynDIG4 and SynDIG4 AVPA, and quantification of the ratio of surface-labeled to total-labeled SynDIG4 for wild-type and AVPA. **(C)** Representative dendrites with wild-type SynDIG4 and SynDIG4 AVPA, and quantification of PSD95 puncta size and colocalization of surface SynDIG4 with PSD95 in wild type and AVPA transfected neurons. To determine the overlap with PSD95, the threshold for each independent experiment was determined by averaging the thresholds from at least 25% of the images. This average threshold was subsequently applied to all images for analysis. Colocalization of SynDIG4 and PSD95 was identified by using a PSD95 mask overlaid on SynDIG4 signals. The amount of SynDIG4 at PSD95 was quantified as the ratio of SynDIG4-PSD95 colocalized puncta to the total number of SD4 puncta. Data are represented as mea*n* ± SEM; *n* = 11–15 cell per group, each group from 2 to 3 independent experiments; ns not significant; *p < 0.05; **p < 0.01; ***p < 0.001; ****p < 0.0001. Scale bar: 5 μm.

We then aimed to assess the role of μ2-dependent SynDIG trafficking within neurons. We transfected mouse dissociated hippocampal neurons at DIV9 with myc-SynDIG1 (WT)-Flag or myc-SynDIG1 (Δ161–76)-Flag constructs and visualized surface and total SynDIG1 using anti-Flag and anti-myc antibodies, respectively, at DIV18. Like our findings in COS cells, overexpression of SynDIG1∆161–76 resulted in an increased surface to total SynDIG1 ratio compared to WT SynDIG1 (Figure 5A). This result further suggests that the SynDIG1-μ2 binding interaction is required for proper endocytosis in neurons and is mediated by a non-canonical endocytic signal.

We conducted similar experiments within rat dissociated hippocampal neurons for SynDIG4 and its μ2-binding impaired mutation, SynDIG4-AVPA. Flag-SynDIG4 (WT)-HA and Flag-AVPA-HA were transfected into rat dissociated hippocampal neurons at DIV0 and visualized at DIV18-22; similarly, anti-HA and anti-Flag antibodies were utilized to compare the surface to total SynDIG4, respectively. Once again, consistent with our results in COS cells, overexpression of AVPA resulted in a higher surface to total ratio of SynDIG4 compared to WT SynDIG4 (Figure 5B). This result also suggests that SynDIG4-μ2 binding is both required for proper endocytosis in neurons and specifically mediated by a canonical YXXɸ signal. Furthermore, AVPA surface puncta were larger than WT SynDIG4 surface puncta, while PSD95 puncta size did not differ between the AVPA and WT SynDIG4 groups (Figure 5C). This result suggests that impaired endocytosis causes SynDIG4 to accumulate on the plasma membrane without affecting the distribution of PSD95. Interestingly, we observed that more PSD95 puncta co-localized with surface AVPA compared to WT SynDIG4, which is primarily found at extrasynaptic sites (Figure 5C). We conclude that the defective endocytosis of AVPA promotes its retention at synapses.

## Discussion

### SynDIG4 colocalizes with GluA1 at the plasma membrane in COS7 cells

We have shown that SynDIG4 has a canonical YXXɸ endocytic signal, and that mutation of this signal disrupts interaction with the μ2 subunit of the AP2 complex, presumably hampering clathrin-mediated endocytosis of SynDIG4 and resulting in its accumulation on the surface of the cell. In transfected heterologous cells, there is almost no observable surface expression of WT SynDIG4 (Figure 2), likely due to rapid endocytosis. The surface localization of the mutant SynDIG4 enabled us to visualize co-localization of SynDIG4 and ionotropic glutamate receptors on the surface of transfected cells (Figure 4). We determined that there is a significantly higher correlation coefficient between SynDIG4 + GluA1 puncta than either SynDIG4 + GluK2 or SynDIG4 + GluA2 puncta. Compared to randomized data, there was also a small but significant difference in SynDIG4 + GluA2 puncta. However, we observed a highly significant difference for GluA1 and no difference for GluK2, suggesting that the correlation coefficient for SynDIG4 + GluA1 is unlikely due to chance and therefore biologically relevant. Whether the preference for SynDIG4 + GluA1 at the cell surface in heterologous cells translates into SynDIG4-mediated regulation of GluA1-containing AMPARs in neurons requires additional experiments beyond the scope of the present study.

It is important to note that in this experiment, because of its higher surface expression, we used the deletion mutant SynDIG4∆170-89-myc instead of the double point mutant AVPA-myc. It is possible that removal of 20 amino acids in the deletion mutant could disrupt an interaction with GluA2. Multiple proteomic studies did find copurification of SynDIG4 with GluA1 as well as GluA2 (Shanks et al., 2012; Schwenk et al., 2012; Chen et al., 2014). Interestingly, in native AMPAR complexes SynDIG4 associates at the interface of GluA1 and another AMPAR auxiliary factor cornichon-2 (Yu et al., 2021). A follow-up proteomics study did report select enrichment of SynDIG4 with GluA1 over GluA2 (Schwenk et al., 2014), consistent with the cryo-EM study. Furthermore, there is reduced extrasynaptic GluA1 and GluA2 in hippocampal neurons derived from SynDIG4 knockout (KO) mice compared with WT (Matt et al., 2018). Together, these data suggest that there is a specific interaction between SynDIG4 and GluA1 (either GluA1 homomers or GluA1/GluA2 heteromers). Additional experiments are needed to understand the relationship between μ2-dependent SynDIG4 trafficking and establishment of extra-synaptic pools of GluA1-containing AMPARs important for synaptic plasticity.

### Disruption of SynDIG interactions with μ2 increase surface expression in cultured hippocampal neurons

In contrast to heterologous cells, we have found that surface expression of both WT SynDIG1 and SynDIG4 is higher in cultured hippocampal neurons. However, neurons did recapitulate the same general phenotype seen in heterologous cells; more specifically, that expression of μ2-binding deficient SynDIG mutants increases surface expression of SynDIG proteins. We found that expression of SynDIG1∆161–76, in comparison to WT SynDIG1, significantly increases surface protein expression in cultured hippocampal neurons. We also observed a significant increase in the surface expression of the SynDIG4 mutant AVPA compared to WT SynDIG4. Intriguingly, higher surface expression of SynDIG4 did not disrupt PSD95 cluster size, though AVPA was found to be co-localized with a higher percentage of PSD95 compared to WT SynDIG4. In future studies, it will be of interest to utilize higher resolution imaging to investigate the sub-synaptic localization of SynDIG4 relative to PSD95 and AMPAR subunits. We have shown using brain fractionation techniques that SynDIG4 is de-enriched from the postsynaptic density (PSD) (Kirk et al., 2016). One possibility is that surface SynDIG4 could be localized extrasynaptically to stabilize pools of GluA1. However, given the role of SynDIG4 in LTD (Troyano-Rodriguez et al., 2019), another intriguing possibility is that SynDIG4 could be localized to endocytic zones (Blanpied et al., 2002; Lu et al., 2007; Petrini et al., 2009), where it could play a specialized role in endocytosis of GluA1-containing heteromers or GluA1 homomers (Sanderson et al., 2016; Sanderson et al., 2021; Sanderson et al., 2012).

We have also shown here that in addition to *μ*2, SynDIG4 will also co-immunoprecipitate with μ1a, μ3a and μ4 (Figure 1). These other AP complexes are involved in a variety of intracellular trafficking events for other AMPAR auxiliary proteins (Guardia et al., 2018). For example, stargazin, an AMPAR auxiliary protein that regulates AMPAR diffusion between extra-synaptic and synaptic sites (Bats et al., 2007), has been shown to possess an endocytic signal capable of interacting with multiple μAP subunits. The region around this signal is differentially regulated by phosphorylation, influencing the binding affinity for different μ subunits and, thus, changes the intracellular trafficking of stargazin and its cargo. The authors showed that mutations in stargazin that disrupt interactions with μ2 and μ3a ultimately impaired trafficking of AMPARs during LTD (Matsuda et al., 2013). However, it remains an open question if phosphorylation or other post-translational modifications affect μAP binding or SynDIG-mediated AMPAR localization important for synaptic function.

### SynDIG1 is enriched in the TGN and traffics to the plasma membrane and early endosomes in COS7 cells

We have shown that SynDIG1 interacts with μ2 through a noncanonical endocytic signal. The deletion mutant myc-SD1∆161–76 does not bind μ2; whereas myc-SD1∆161–72 does, suggesting that the critical amino acids could be FLMM (YXXɸ replacing Y with F), which is not unprecedented (Al-Hasani et al., 2002; Hu et al., 2001). It is worth noting that the myc-SD1∆161–72 construct expressed at a lower level than other constructs, making co-immunoprecipitation comparisons more challenging. Nonetheless, when normalized for the lower expression, the amount co-immunoprecipitated was comparable to WT (Figure 1C). To make matters more complicated, SynDIG2, which is highly similar to SynDIG1 in this region, does not bind μ2 nor any μAP subunit tested (Figure 1B). Further mutation analysis, including the construction of SynDIG1/SynDIG2 chimeras, will be helpful in clarifying these complications.

When we blocked protein translation with cycloheximide to investigate steady state localization, we observed that both SynDIG1 and SynDIG2 are localized to the Golgi apparatus, which had been noted previously for SynDIG2 (de Chaldée et al., 2006), and to early endosomes. We showed that both molecules are enriched at the TGN rather than the cis-Golgi (Figure 3). Deletions of roughly homologous portions of each protein (SynDIG1∆161–76 and SynDIG2∆135–55) resulted in increased surface labeling (Figure 2) and decreased overlap with the TGN marker Golgin-97 (Figure 3). Using an antibody feeding approach with a myc-SynDIG1-Flag construct, we observed internalized SynDIG1 signal that overlaps with Golgin-97 (Figure 3D).

Taken together, these data show that both SynDIG1 and SynDIG2 are similar in that they are localized to the TGN, that they are present in early endosomes, and that they can traffic to the plasma membrane. However, they appear to be endocytosed through different mechanisms: SynDIG1 interacts with μ2, whereas SynDIG2 does not. One alternative explanation is that either or both molecules could harbor a Golgi retention signal in the deleted region (Banfield, 2011), the removal of which could result in egress from the Golgi. For instance, disruption of a hypothetical Golgi retention signal in the SynDIG2∆135–55 mutant, could explain the accumulation of this mutant on the plasma membrane without requiring disruption of a μ2 binding interaction (Figure 2B).

We have also shown that SynDIG1 interacts with the μ1a subunit (Figure 1C). The AP1 complex can mediate both forward trafficking from the TGN to endosomes and retrograde transport of cargo from endosomes to the TGN (Guardia et al., 2018; Tu et al., 2020; Farias et al., 2014; Progida and Bakke, 2016). In this manner, SynDIG1 trafficking is similar to TGN38, an integral membrane protein that requires a YXXɸ signal to cycle constitutively between the TGN and the plasma membrane (Roquemore and Banting, 1998). However, the possibility remains that even though SynDIG1 interacts with μ2 and μ1a, some other molecular machinery which interacts with residues 161–76 could control trafficking to and from the TGN.

How might SynDIG1’s localization and trafficking impact synapse development and plasticity? We speculate that SynDIG1 could be involved in sorting or trafficking of specific cargo in neurons during synapse development. In response to activity blockade, SynDIG1 might also alter trafficking of cargo, or posttranslational modifications such as palmitoylation could inhibit its interaction with AP2 and cause it to accumulate at the cell surface (Kaur et al., 2016) where it serves a direct role in synapse function. Future experiments are needed to address these questions regarding the intracellular trafficking of SynDIG1.

Taken together, our results provide insight into the mechanisms by which SynDIG proteins are targeted to intracellular compartments via an μAP-dependent manner. These foundational studies represent a first step in understanding SynDIG-mediated establishment of extra-synaptic pools of AMPARs important for synaptic plasticity. Many studies highlight the importance of dynamic AMPAR trafficking to and from synapses in the rapid modulation of synaptic strength during plasticity. Additional experiments in hippocampal neurons, including super-resolution and live-imaging approaches, are needed to expand upon these present results in the context of synaptic plasticity and are the focus of our current research efforts.

## Data Availability

The raw data supporting the conclusions of this article will be made available by the authors, without undue reservation.
